# Resource availability and human activity shape the landscape distribution of white rhinoceros, a highly threatened African megaherbivore

**DOI:** 10.1007/s00442-025-05845-7

**Published:** 2026-02-05

**Authors:** Emilia S. M. Staegemann, Timothy Kuiper, Dave J. Druce, Graham I. H. Kerley, Siphesihle Mbongwa, Joris P. G. M. Cromsigt

**Affiliations:** 1 Centre for African Conservation Ecology, Nelson Mandela University, Gqeberha, South Africa; 2 Department of Conservation Management, Nelson Mandela University, George, South Africa; 3 Research Department, Welgevonden Game Reserve, Vaalwater, South Africa; 4 Ezemvelo KZN Wildlife-Scientific Services, Pietermaritzburg, South Africa; 5 Department of Wildlife, Fish and Environmental Studies, Swedish University of Agricultural Sciences, Umeå, Sweden; 6 Copernicus Institute of Sustainable Development, Utrecht University, Utrecht, The Netherlands

**Keywords:** Megaherbivore concept, Animal space use, Edge effect, Predator–prey interactions, Poaching, Ecology of fear

## Abstract

The megaherbivore concept suggests that mammals >1000 kg are insensitive to predation as adults. Consequently, their space use should be largely driven by resources. This does not account for the fact that megaherbivores have been hunted by humans for >100,000 years and likely evolved innate responses against human predation. Recent studies indeed show that megaherbivores, such as elephants and rhino, strongly respond to human voices. Few, however, have examined the relative influence of resource versus human risk drivers on the landscape use of megaherbivores. Using a long-term dataset from aerial rhino surveys and poaching events in Hluhluwe-iMfolozi Park, South Africa, we investigated how resource and human risk factors shape the landscape distribution of white rhinoceros (*Ceratotherium simum*). We used rainfall, fire, catena position, and terrain ruggedness as resource drivers and poaching intensity, and distance to human sound-generating infrastructure as human risk drivers. Both resource and human risk drivers affected rhino landscape distribution. Rhino preferred valley bottoms over midlands and uplands, and the use of the latter two habitats increased during the dry season. During drier wet seasons, rhinos increased their upland habitat use. Rhino avoided the park’s fenceline and other infrastructure (roads and camps). Poaching intensity did not influence rhino landscape use. Avoidance of human infrastructures may reduce the effective size of protected areas for rhino. Future work should assess how rhino respond to resources and risk over shorter timescales. Our findings encourage a re-evaluation of the megaherbivore concept to include humans as drivers of their ecology.

**Supplementary Information:**

The online version contains supplementary material available at https://doi.org/10.1007/s00442-025-05845-7.

## Introduction

Optimality theory predicts that animals use their environment in a way that maximizes benefits and reduces their costs, thereby increasing fitness (Parker and Smith 1990). This may lead to a trade-off between finding optimal resources (e.g. food, mates, and water) while reducing predation risk (Hopcraft et al. 2010), with these drivers determining the space use of animals across the landscape by creating so-called “foodscapes” (Searle et al. 2007) and “landscapes of fear” (Laundré et al. 2010). These concepts describe how animals perceive, and subsequently respond to, spatial variation in predation risk (landscape of fear) and in the quantity and quality of resources (foodscape) across landscapes. Body size influences the relative importance of resources and risk factors as drivers of animal landscape use, because larger prey species are less vulnerable to predation but need more food (Hopcraft et al. 2010). Therefore, larger herbivores tend to be driven more by the need to locate resources than to avoid predation (Hopcraft et al. 2010). This may be particularly the case for so-called “megaherbivores”, mammalian terrestrial herbivores exceeding >1000 kg in body mass as adults (Owen-Smith 1988). Due to their very large body mass, they are hypothesized to be largely immune to predation as adults (Owen-Smith 1988), and their behaviour and landscape use is thus hypothesized to be largely driven by the availability and distribution of resources. Megaherbivore calves and juveniles, however, can be susceptible to predation (see, e.g. lion predation on elephant (Loveridge et al. 2006) and black rhino calves (Brain et al. 1999)), and both elephant and rhino actively defend their young against large carnivores (McComb et al. 2011).

This view on resources as the main driver of megaherbivore behaviour is based on non-human predation. However, megaherbivores have faced the threat of hunting by hominins for millions of years (Lyons et al. 2004; Surovell et al. 2005), and despite this, they have persisted in African and Asian landscapes (Owen-Smith 1987). This relates to the concept of humans as a super-predator, which states that human predation has shaped the evolutionary and ecological responses of a very large number of prey species globally (Darimont et al. 2015). It is, therefore, reasonable to expect that this threat of human predation led to adaptive anti-human predation behaviour among extant megaherbivore species (c.f. Cromsigt et al. 2013). From the late 1800s to the early 1900s, human hunting on most megaherbivores became illegal following strict conservation measures. Subsequently, megaherbivore populations in southern Africa experienced none to very little threat from human hunting for almost a century. During this time, live capture of megaherbivores, such as the white rhinoceros (*Ceratotherium simum*, hereafter “rhino”), for translocation among protected areas developed and became regular practice from the 1960s. Due to the prevailing view that megaherbivores are largely predation insensitive, very few studies have investigated the relative roles of resource (food, water) versus human risk drivers on their behaviour. The super-predator concept, however, tells us that it is unlikely for megaherbivores to have lost their innate anti-human predation responses over the last hundred years of conservation. This lack of understanding has become a particular concern for the white rhino since 2008 when southern Africa started experiencing a serious rhino poaching crisis (Biggs et al. 2013). Here, we therefore used a unique long-term dataset on white rhino distribution in Hluhluwe-iMfolozi Park, South Africa, to assess if and how white rhino occurrence responds to resource versus human risk drivers during a period when poaching increased from nearly absent to intense poaching.

The original and pivotal work of Norman Owen-Smith suggests that rhino move seasonally between different grassland types linked to specific landscape positions (Owen-Smith 1973), moving from grazing lawns in the flat valley bottoms during the wet season to *Dactyloctenium* and *Panicum* grasslands in riverine woodlands during the early dry season, and taller *Themeda* grasslands on the midlands to crests of hills in the late dry season. During later work, Owen-Smith and colleagues showed how rainfall mediates this seasonal pattern and how rhino spatial and temporal landscape use may vary between years depending on the amount of rainfall. During high rainfall years, rhino may focus on short grass lawns into and even throughout the dry season, whereas during dry years rhino may move to the woodland grasslands and hillslope *Themeda* grasslands already during the wet season (Shrader et al. 2006; Arsenault and Owen-Smith 2011). In addition to rainfall, fire may also influence how rhino use the landscape through its influence on the productivity and nutritional value of grasslands (Archibald et al. 2005). Previous work has suggested that rhino favour green grass regrowth in recently burnt *Themeda* grasslands, particularly during the dry season (Shrader et al. 2006). Since fire and rainfall both influence grass productivity (Archibald 2008), they likely also interact in driving the local distribution of rhino, but this remains to be tested.

Very little work has been done on how human risk drives rhino movements and distribution. However, the super-predator concept suggests that human scent and voice are the most likely cues to trigger fear responses (Darimont et al. 2015; Zanette et al. 2023). Indeed, independent experimental studies in two different South African areas showed that rhino responded strongly to human voices relative to large carnivore (lion) and control sounds (Nhleko et al. 2022; Zanette et al. 2023). In the study by Nhleko et al. (2022), female white rhino decreased their visitation to experimental plots by 70% following human voices. Interestingly, males did not reduce their visitation, but both sexes increased their vigilance in response to human voices. Using 5 years of aerial survey data, le Roex et al. (2020) showed how the occurrence of white rhino in Kruger National Park decreased with proximity to human sound-associated infrastructure, such as reserve fence lines, camps, and roads. All this work suggests that rhino do indeed exhibit human avoidance behaviour, especially in relation to human sounds, presumably as an evolved innate response to the human super-predator.

One relevant aspect that remains to be tested is whether rhino respond behaviourally to spatial poaching hotspots, i.e. whether they can pick up cues alerting them to reduce their use of these risky hotspots, similar to their avoidance of human infrastructure, as shown by le Roex et al. (2020). Poaching is different from the above-mentioned human cues since modern poaching typically happens away from human infrastructure in a quiet, signal-poor manner where they kill rhino without causing olfactory, sound, or visual risk cues. This has, in fact, led authors to speculate about poaching as an ecological trap, where rhino are unaware of the fact that the resource-rich areas to which they are attracted also attract poachers, resulting in increased risks that rhino are unaware of due to an absence of risk signals (le Roex et al. 2020). The conclusion of this work is, therefore, that, in contrast to human sound-inducing activities and infrastructure, rhino are not likely to respond behaviourally to poaching risks. However, if poaching happens consistently in the same area from year to year, creating clear predictable poaching hotspots for surviving rhino in that area (i.e. through them frequently encountering dead rhino and/or losing family members), it is not unrealistic to assume that rhino learn to avoid such areas.

We are unaware of studies testing how resources and human risk drive rhino behaviour and occurrence patterns simultaneously. Incorporating both is essential, because predator–prey theory tells us that the risk responses of prey depend on, and vary with, resource availability. A classic work by Joel Brown showed how prey animals must balance two essential pressures: they must find and eat food without being eaten (Brown et al. 1999). This also means that prey are more likely to respond to risk during times when, and in places where, resources are not limiting. In this study, we therefore asked how rhino deal with the trade-off between finding resources and avoiding human risk. We investigated distribution responses of rhino to sound-generating infrastructure as well as spatially predictable poaching hotspots using 10 years of seasonal rhino distribution data from bi-annual white rhino aerial surveys in Hluhluwe-iMfolozi Park (HiP). We linked rhino occurrence to spatially explicit data on drivers of resource availability/accessibility and on human risk drivers. Resource indicators included landscape catenal position, rainfall, and fire—all influencing forage quantity and quality—as well as landscape ruggedness, which affects resource accessibility. Risk indicators encompassed poaching incidence and the presence of human sound-associated infrastructure. We predicted that rhino will:

- select areas in the flat valley basin of HiP during the wet season and midlands (i.e. midslopes) and uplands during the dry season, tracking the phenology of their grass resources (following Owen-Smith 1973). We also predicted that rainfall conditions would shape this pattern, and that the selection for midlands and uplands during the dry season would be less strong during relatively wet years, when grass resource availability in the basin remains higher even in the dry season, while the selection for the basin during the wet season would be less strong during relatively dry years, when grass availability in the basin is depleted more quickly (following Shrader et al. 2006 and Arsenault and Owen-Smith 2011).
- avoid rugged terrain due to the difficulty of moving through such areas to access resources (following le Roex et al. 2020).
- select burnt areas within 1–2 months of fire (following Shrader et al. 2006), when grass regrowth in the burnt areas provides a high-quality food resource. We predict that rainfall will influence this effect, where high rainfall during the months following the burning would increase the selection of burnt areas by rhino because of the rain promoting the extent of grass regrowth in the burnt areas.
- avoid areas close to human sound-associated infrastructure (fencelines, roads, camps) (following le Roex et al. 2020; Nhleko et al. 2022; Zanette et al. 2023). We also predict this avoidance to be stronger for infrastructures associated with more, or more frequent, sounds (i.e. stronger response to busy tourist roads than to management tracks, stronger response to noisier camps).
- avoid poaching hotspots (i.e. areas with high poaching intensity year after year). Alternatively, rhino will not avoid these hotspots because of an absence of risk cues in these areas. In this latter case, these hotspot areas could represent ecological traps (sensu le Roex et al. 2020).

## Materials and methods

### Study area

Hluhluwe-iMfolozi Park (HiP) is situated in the KwaZulu-Natal Province of South Africa between S 28.0000 and 28.4300, and E 31.7160 and 32.015, with an area of 92 657 ha. Altitude ranges between approximately 45–750 m above sea level (masl—Howison et al. 2017). The annual rainfall ranges from 990 mm in the north of the Park (Hluhluwe) to less than 635 mm in the south (iMfolozi), with the highest rainfall occurring during the wet summer season from November to April and the lowest during the dry winter season from May to September (Balfour and Howison 2002). The vegetation in the park varies from open, parkland-type savanna to more closed woodland savanna and includes both fine-leafed and broad-leafed dominated communities. Other vegetation types include mist-belt forests and grasslands on the crests and top slopes of the higher hills, and thicket communities consisting of dense shrubs (Whateley and Porter 1983). This study was conducted in the iMfolozi section where the HiP rhino aerial censuses are conducted. The entire park is fenced, with a public road traversing between the Hluhluwe and iMfolozi sections.

### Rhino location data

Annual aerial census surveys of rhino in the iMfolozi section of the park have been conducted since 2008. From 2014 onward, this was changed into bi-annual counts, one census in the wet season. These surveys are full-coverage counts from a fixed-wing aircraft, flying parallel, adjacent transects at 250 ft (80–100 m) above the ground and with a constant speed, following line transects. The transects are spaced 500 m apart, and three observers and a pilot count rhino up to 250 m on either side of the aircraft. For each sighting of a rhino, they record the number of animals, the age class of each individual, and the location data (GPS coordinates). The census is completed in approximately 3 days, with a duration of approximately 3–3.5 h per day in the midmorning while animals are active, to reduce the risk of double counting due to large-scale movements of the rhino. The data are captured on a laptop using CartaLinx software that updates in real time. This is an important feature that helps avoid double counting animals. Experienced observers keep track of the locations of all observed rhino on the Cartalinx map and by using natural features such as hills and rivers. For example, if a rhino is observed 250 m in on the left side on one transect and then approximately 250 m in on the right side on the parallel reverse transect in approximately the same location, it is considered the same individual. For the analyses presented in this study, we used a total of 6,632 rhino observations between 2012 and 2023. In addition to the risk of double counting, there is also a risk of individuals being missed during the aerial surveys, but the above-described systematic procedure aims to mitigate this risk of underdetection of rhino as effectively as possible.

As the rhino locations are recorded along transects from a moving aircraft, it is difficult to pinpoint the exact position of the rhino, especially since there is a slight delay between the time of observing a rhino and the time when the coordinates are captured. To account for the uncertainty in the rhino locations, predictor rasters were generated using a 500 by 500 m resolution. Raster values were then extracted to the rhino GPS locations.

### Estimation of predictor variables

#### Catena position and terrain ruggedness

A digital elevation model (DEM) with a spatial resolution of 20 by 20 m and a slope raster file with a spatial resolution of 20 by 20 m were concatenated to create a combined raster with elevation and slope. The combined raster was reclassified into three catena category values: basin (<200 masl or slope <7%), midlands (200–300 masl or slope >7%), and uplands (>300 masl). The catena position for each rhino observation was then extracted from this raster. Terrain ruggedness was calculated from the digital elevation model using the terrain ruggedness index tool in QGIS.

#### Rainfall and fire

Rainfall was recorded daily using a manual rain gauge and summed at the end of each month at three different rain stations in iMfolozi (Makhamiza, Mbuzane, and Masinda) during the period of our study. Due to occasional missing data for some months, monthly rainfall was averaged across the three stations over the 6 months preceding each census count and then categorized as “low” or “high” rainfall for that census. One median rainfall value for the whole study period (2012–2023) per season served as a threshold, with values below the median classified as ‘low’ and those above as “high” for each season separately.

Fire data were extracted using historical (2011–2022) yearly fire point maps from MODIS. The points were converted into polygons to standardize which areas had been burnt. A grid cell layer of 500 × 500 m was created, where the time since fire in each grid cell was counted for the time of each rhino census count. Grid cells that covered less than 50% of a burnt area were excluded from the analysis, as they may not have the same ecological impact as grid cells that were fully burnt. Time since fire (months) was counted in the grid cells for each year and season from the date of each of the rhino census counts, respectively. Based on this, grid cells were categorized as burnt recently, burnt the previous season, and unburnt (burnt more than 7 months prior to the counts).

#### Human risk and disturbance variables

We received data on illegal (poaching) and legal (management) removals, including dates and GPS locations for all individual removals (EKZNW unpublished records). Legal, live removals of rhinos occur a few times a year during certain years whereby rhinos are relocated to other reserves. This management strategy follows a source–sink approach, where rhino are removed from high-density sink areas to allow for natural dispersal of rhino from surrounding source areas (Linklater and Shrader 2017). Illegal and legal removal locations during the 12 months prior to each rhino count were used to create removal density maps using kernel density smoothing with a resolution of 500 × 500 m. We used these removal density maps as a proxy for human “predation risk”.

Using the distance function from the terra package in R, we calculated the distance from the rhino locations to the nearest boundary fence, staff and tourist camps (larger and smaller), tourist roads, and management tracks. This resulted in a raster of 500 × 500 m where each cell’s value represents its distance to each of the variables, representing potential human disturbance.

#### Statistical analysis and modelling approach

Data from each predictor variable was extracted to the rhino locations recorded bi-annually from 2012 to 2023. We used resource selection functions (RSF) comparing the values of environmental and risk variables at rhino location points with values for these variables at random locations, following the generation of ten random points for each rhino location (Barbet-Massin et al. 2012). RSF was run as generalized linear mixed models using the R package lme4 with rhino occurrence as a binomial response variable (1 for rhino presence locations and 0 for the randomly generated available locations). Because our background (“0”) locations were generated randomly across the study area to represent what was available to rhinos, they are not true absences, and logistic regression under this sampling design does not yield true probabilities of presence (Keating and Cherry 2004). Therefore, following the recommendation of Pearce and Boyce (2006), we refer to model outputs as the relative probability of rhino presence, rather than true probabilities. The predictor variables included drivers of food resource quality and availability (catena position, time since fire, rainfall) or food accessibility (terrain ruggedness) and human risk drivers (illegal and legal removal kernel density, and distance to boundary fence, tourist roads, staff and tourist camps, and management tracks).

Predictor variables were tested for multicollinearity before running the models using the variance inflation factor (VIF), which showed no evidence of problematic multicollinearity (see appendices). We extracted the fitted values and residuals, generating residual plots which we examined for non-linearity and heteroscedasticity (Fig. 5). These confirmed that the assumptions of normality of errors and variance were met. To assess the predictive performance of the models, the area under the curve (AUC) of the receiver operating characteristic (ROC) curve was calculated (Hanley and McNeil 1982), and all the predictors were retained.

We initially included water availability, but this variable was removed to avoid confounding effects since water availability was strongly correlated with catena position. We kept catena position as it explained more variation than water availability. To test our hypothesis that rainfall (wet versus dry years) affects seasonal selection of catena positions, we included the interaction between catena position and rainfall. In a separate model and then tested for the effects of fire. We had to model fire and rainfall separately because the data in this analysis were split between wet and dry seasons. All fires in the park occur during the dry season, so the recent fires in relation to rhino presence only occur during the dry season immediately prior to the dry season counts, while the “fire during the previous season” predictor can only occur during the wet season. In an additional analysis, we split the dataset into two sets of years, 2012–2016, representing years with low poaching activity, and 2017–2023, representing years with high poaching activity. We ran these models for these two periods to test whether the effects of risk variables differed between periods with low versus high poaching activity. In all models, we included rhino census count as a random effect.

## Results

### Model with resource and human risk drivers

**Fig. 1:**
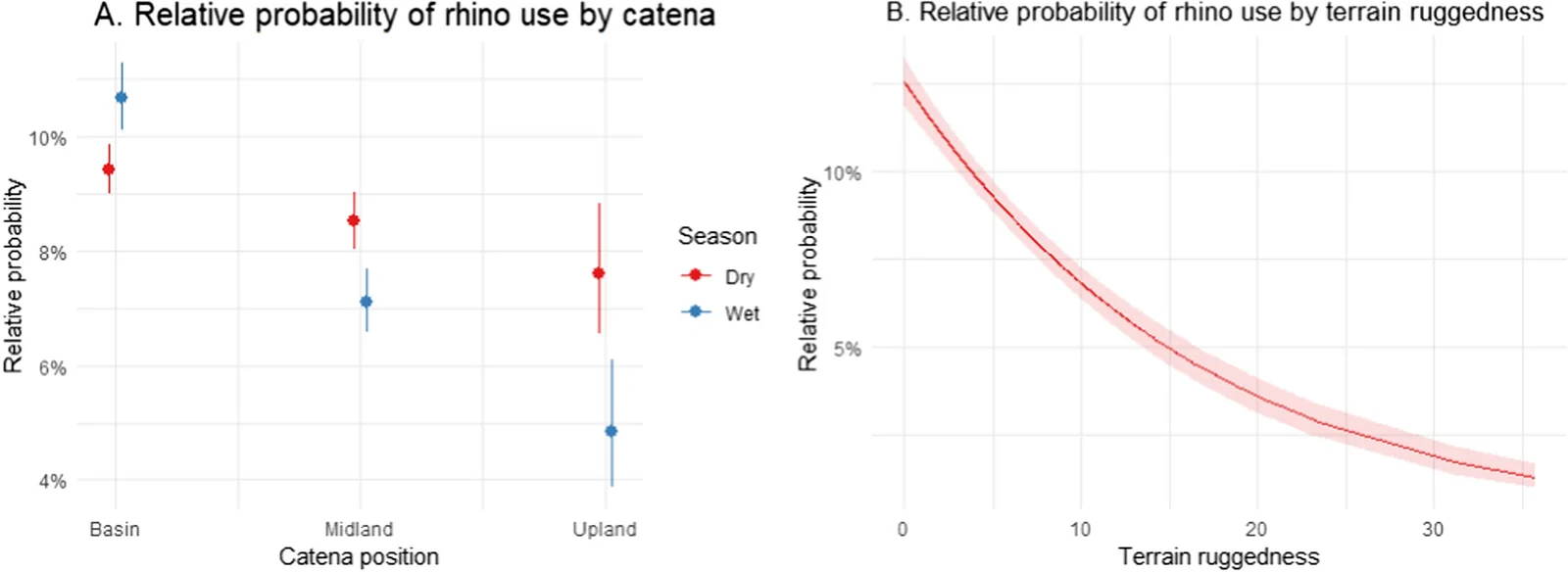
(**A**) Rhino presence increases in basin and decreases on midlands and uplands during the wet season compared to the dry season. (**B**) Rhino presence decreases in rugged terrain

**Fig. 2:**
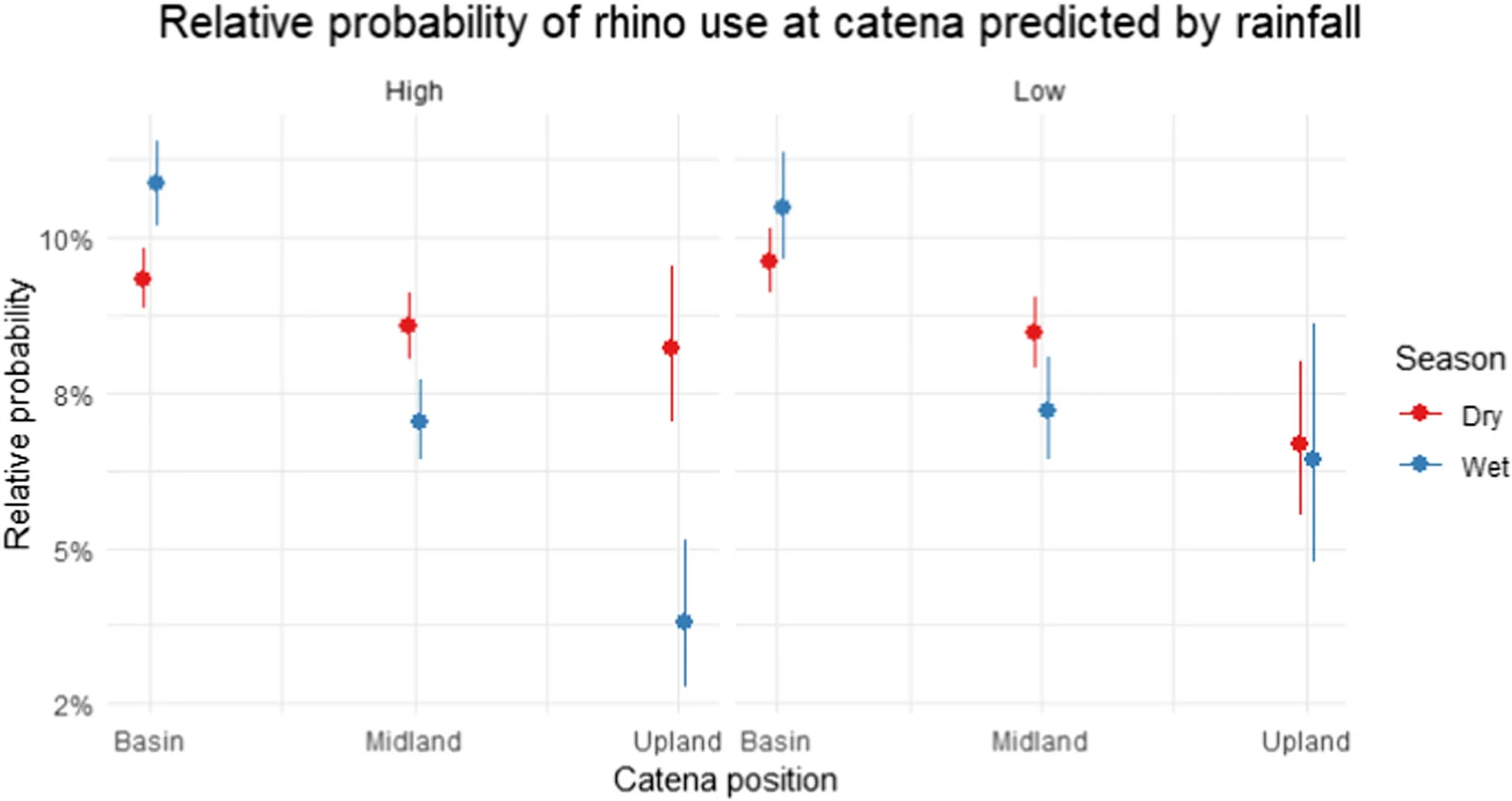
(Left) Rhino presence increases on uplands ad decreases on midlands and uplands during the wet season compared to the dry season during periods with high rainfall. (Right) Low rainfall periods do not significantly affect rhino presence on any of the catena positions

Overall, across all years, the likelihood of rhino presence was highest in the basin habitat, followed by the midlands and then upland habitats (Fig. 1A, Table 1). There was no significant difference in rhino presence between upland and midland *(p* = *0.17, SE* = *0.09, z* = *−1.37).* There was a strong seasonal effect on the use of catena position, where the probability of finding rhino in midlands and, especially, upland areas was higher during the dry than during the wet season, while the probability in the basin was lower during the dry than wet season (Fig. 1A, Tables 1 and 2). The likelihood of rhino presence decreased strongly with increased terrain ruggedness (Fig. 1B, Table 1). Rainfall influenced the seasonal use of catena positions, with the use of uplands being similar in both wet and dry seasons during relatively dry years, but not during relatively wet years when the use of uplands was much lower during the wet season (Fig. 2, Table 1).

**Table 1:** Estimate table for the model including human risk and resource predictors

	Estimate	Std. error	*z*-value	*p*-value
Intercept	−2.545	0.066	−39.415	<0.001***
Distance to management tracks	4 −0.000	10.000	−2.564	0.010*
Distance to big camps	0.000	0.000	2.83	0.005**
Distance to small camps	0.000	0.000	5.189	<0.001***
Distance to the boundary fence	0.000	0.000	11.438	<0.001***
Distance to road	0.000	0.000	1.845	0.065
Poaching intensity	0.033	0.058	0.569	0.569
Legal removal intensity	0.314	0.074	4.211	<0.001***
Midland	−0.094	0.046	−2.060	0.039*
Upland	−0.126	0.089	−1.415	0.157
Wet season	0.165	0.046	3.596	<0.001***
Low rainfall	0.015	0.043	0.347	0.728
Ruggedness	−0.069	0.004	−15.812	<0.001***
Midland: wet season	−0.380	0.077	−4.946	<0.001***
Upland: wet season	−0.996	0.190	−5.251	<0.001***
Midland: low rainfall	−0.036	0.070	−0.540	0.589
Upland: low rainfall	−0.259	0.139	−1.869	0.062
Wet season: low rainfall	−0.641	0.072	−0.890	0.373
Midland: wet season:low rainfall	0.100	0.120	0.828	0.407
Upland: wet season:low rainfall	0.853	0.275	3.102	0.002**
Area under the curve value: 0.60			AIC: 43710.69

**Table 2:** ANOVA table for the model with resource and risk predictors. Terrain ruggedness explains variation in rhino distribution the most (highest *Χ*^2^ value), followed by distance to the boundary fenceline

	*Χ*^2^	Df	*p*-value
Distance to management tracks	6.576	1	0.010*
Distance to big camps	8.011	1	0.004**
Distance to small camps	26.931	1	<0.001***
Distance to boundary fence	130.817	1	<0.001***
Distance to road	3.404	1	0.065
Poaching intensity	0.323	1	0.569
Legal removal intensity	17.733	1	<0.001***
Catena	92.096	2	<0.001***
Season	0.011	1	0.915
Rainfall	0.168	1	0.681
Ruggedness	250.012	1	<0.001***
Catena: season	44.969	2	<0.001***
Catena: rainfall	0.115	2	0.943
Catena: season:rainfall	9.742	2	0.008**

**Fig. 3:**
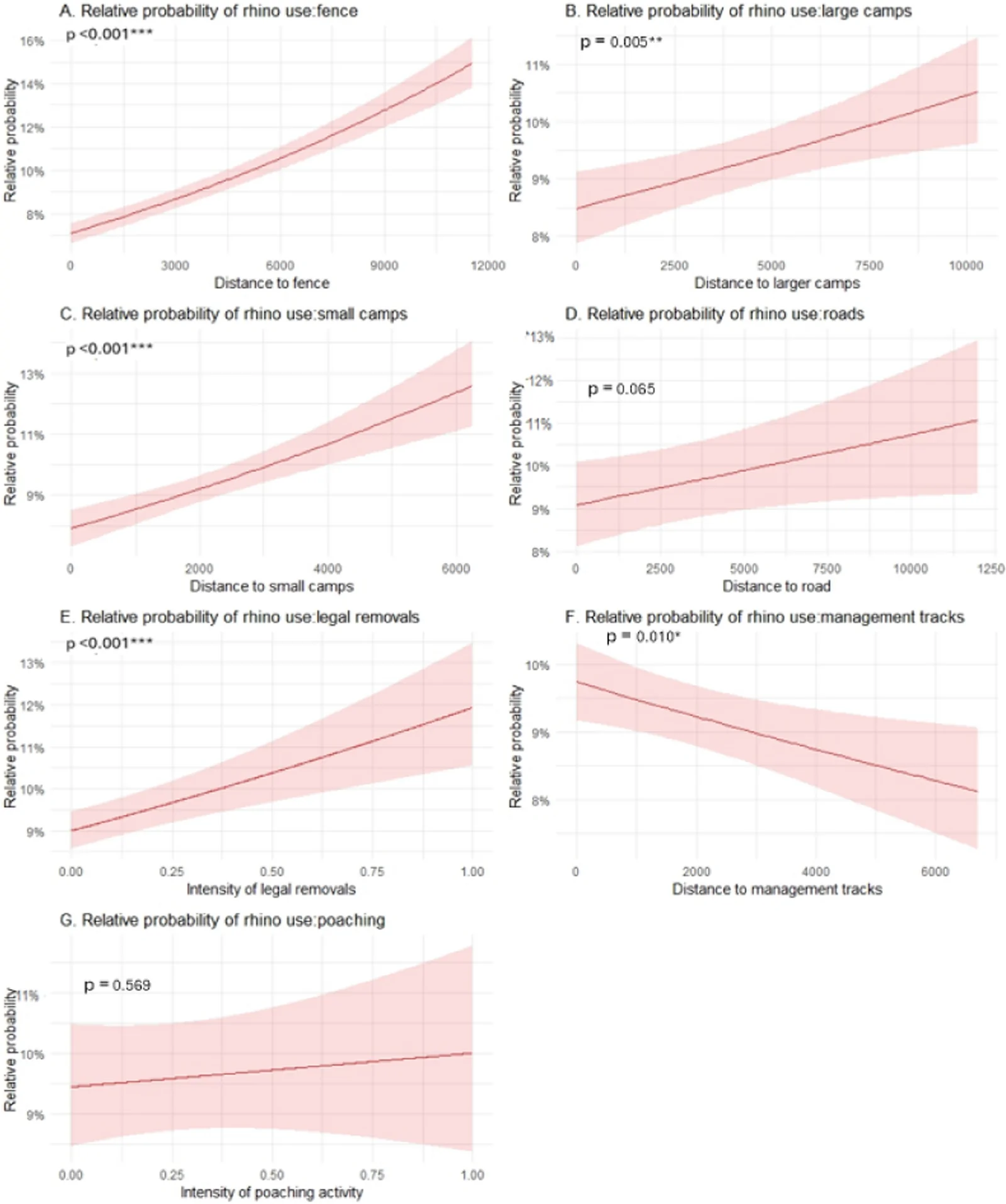
(**A–C**): The probability of rhino presence increases significantly with distance to camps and the boundary fenceline. (**D**) Distance to roads does not explain variation in rhino presence. (**E**) Rhino presence increases with increased intensity of legal removal activity. (**F**) Rhino presence decreases with distance to management tracks. (**G**) Poaching intensity does not explain variation in rhino presence across the landscape

In addition to these resource drivers, several human risk variables affected the likelihood of rhino occurrence. The likelihood of rhino occurrence decreased with distance to staff and tourist camps, the boundary fence, and public roads, although only marginally significant for the latter (Fig. 3 A–D, Table 1). The likelihood of rhino presence was higher in areas with a high intensity of legal removals (Fig. 3E) and close to management tracks (Table 1). Poaching intensity did not influence the probability of rhino presence (Fig. 3G). Interestingly, the likelihood of rhino presence increased closer to management tracks (Fig. 3F). The additional models using low and high poaching data separately showed the same results as the model for all years combined, suggesting there were no differences in the effects of resource and human risk drivers between low and high poaching periods (*see appendices*).

### Fire and rainfall

**Fig. 4:**
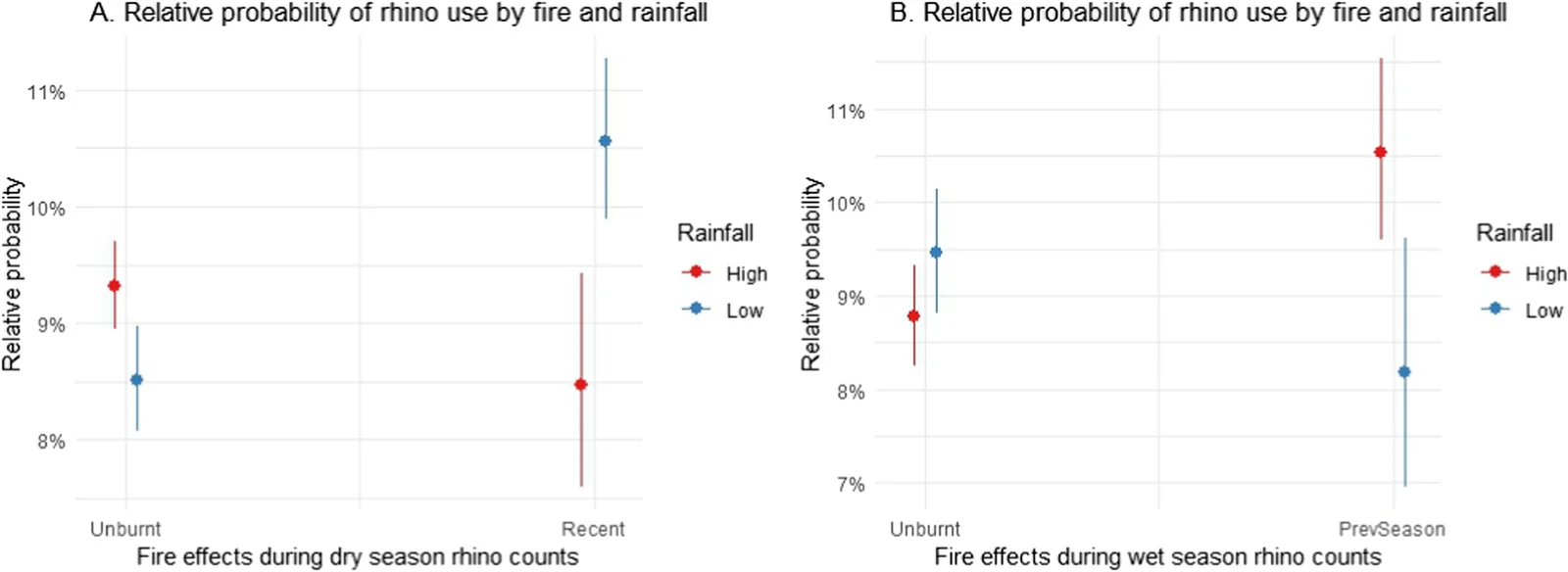
(**A**) Rhino presence increases in recently burnt areas during low rainfall compared to high rainfall during dry season and increases in unburnt areas when rainfall is high compared to low during dry season. (**B**) Rhino presence increases in areas burnt the previous season during high rainfall compared to low rainfall during wet season and increases in unburnt areas when rainfall is low compared to high during wet season

Burnt areas attracted rhino, but effects on their likelihood of occurrence varied with season and rainfall amount (Tables 3 and 4, Fig. 4). During the dry season, the likelihood of rhino occurrence was higher on recent burns relative to unburnt areas, but only during low rainfall years (Fig. 4A). During the wet season, the likelihood of rhino occurrence in areas burnt during the previous dry season 5–7 months ago was higher than that in unburnt areas, but only during high rainfall years (Fig. 4B).

**Table 3:** ANOVA table showing which predictors explain rhino movement when analysing the impact from fire and rainfall during the wet season

	*Χ*^2^	Df	*p*-value
Fire	9.332	1	0.003*
Rainfall	0.548	1	0.459
Fire:rainfall	18.217	1	<0.001***

The interaction between fire and rainfall explains the most variation

**Table 4:** ANOVA table showing which predictors explain rhino movement when analysing the impact from fire and rainfall during the dry season

	*Χ*^2^	Df	*p*-value
Fire	3.513	1	0.060
Rainfall	0.055	1	0.813
Fire:rainfall	9.631	1	0.002**

The interaction between fire and rainfall explains the most variation

## Discussion

Our findings confirm the original work of Owen-Smith (1973) showing that the likelihood of rhino occurrence was higher in the basin than on midlands and uplands, especially during the wet season. Our findings, also, confirm follow-up studies by Shrader et al. (2006) and Arsenault and Owen-Smith (2011) that the rainfall during a given wet or dry season influences rhino’s seasonal use of the catena. Thus, we found that during relatively low rainfall wet season years, rhino increased their use of upland areas during the wet season (Fig. 2). The top three explanatory variables included both resource and human risk drivers: terrain ruggedness, distance to the boundary fence, and catena position. Following this, rhinos are more likely to occur in the basin on flatter terrain and away from the boundary fence. The *χ*^*2*^ values (Table 5) suggest that these three variables explained four to ten times more variation in the likelihood of rhino occurrence than any of the other variables. Rhino also avoided camps and public roads, although these variables explained much less variation than the top three variables. Interestingly, we found some indication for rhino likelihood of occurrence to be higher close to management tracks. Below, we discuss these findings in more detail.

**Table 5:** Table showing estimate values for the model with recent fire as the predictor

	Estimate	Std. error	*z*-value	*p*-value
Intercept	−2.275	0.023	−98.858	<0.001***
Recent fire	−0.105	0.064	−1.627	0.103
Low rainfall	−0.098	0.037	−2.645	0.008**
Recent fire and low rainfall	0.343	0.080	4.268	<0.001***
Area under the curve value: 0.52			AIC: 29,558.62	

It is important to emphasize that the area under the curve values (AUC) for both models resulted in values as low as 52% (see tables in appendices). This means that our models still contained a lot of unexplained variation in rhino landscape distribution. Such variation may be due to missing ecological factors that are important for explaining rhino landscape use, such as the potential roles of other species in the landscape (e.g. elephants, Slotow et al. 2001) or variation in food availability not captured by our coarse measure of catena position. Additionally, the coarseness of several of our datasets (e.g. fire and rainfall) may also have reduced the statistical power of our models. In addition to our model fits, we also need to highlight the relatively small effect sizes of many of our predictor variables, suggesting that the practical implications of our findings may be limited despite their statistical significance (Table 6).

**Table 6:** Table showing estimate values for the model with fire from the previous season as predictor

	Estimate	Std. error	*z*-value	*p*-value
Intercept	−2.342	0.033	−69.316	<0.001***
Previous season fire	0.202	0.062	3.245	0.001**
Low rainfall	0.083	0.052	1.597	0.110
Previous season fire and low rainfall	−0.361	0.116	−3.103	0.002**
Area under the curve value: 0.53			AIC: 14,685.45	

### Fire and rainfall

High dry season rainfall on recently burnt areas did not increase the probability of rhino presence, as was hypothesized. On the contrary, high rainfall periods had lower proportions of rhinos in recently burnt areas compared to low rainfall periods. A possible explanation for this pattern could be that rhino move to grazing lawns when rainfall is higher (Owen-Smith 1988), and these areas are often unburnt due to the lack of fuel load, leading to a decrease of rhino in nearby burnt areas. We found a higher probability of rhino presence in areas burnt in the previous season, 5–6 months before the wet season count. Since fires only occur during the dry season in iMfolozi, this may reflect an attraction of rhino to burnt areas 5–6 months during the following wet season. This is supported by our finding that higher wet season rainfall increased rhino presence in the areas burnt the previous season. We did not find support for the hypothesis that recently burnt areas (burnt 2–3 months ago) with a fresh growth of nutritious grass would attract rhino the most (following Shrader et al. 2006). However, our coarse data and approach (counts every 6 months and poaching density aggregated at 500 by 500 m scale) in combination with varying timing of dry season burns among years may very well have masked relatively short-term and finer spatial scale responses of rhino to dry season burns.

### Effects of human risk and disturbance variables

Our findings confirmed our hypothesis that rhino would respond to human sound-generating infrastructures because of innately evolved responses against human predation and human voices as risk cues. We found a reduced rhino likelihood of occurrence closer to the reserve fenceline and large and small camps. We also found weak evidence for a negative effect of tourist roads on rhino likelihood of occurrence. Our findings confirm other recent studies showing that rhino strongly respond to human sounds (Nhleko et al. 2022; Zanette et al. 2023) and infrastructures (le Roex et al. 2020). Interestingly, we found that rhino responded more strongly to smaller than to large camps: the distance to small camps explained more variation and its effect on rhino occurrence was stronger than for the distance to large camps. The large camps in our analysis were mainly tourist camps, whereas the small camps were field ranger camps. We speculate that the stronger response to ranger camps may be due to these camps generating more audible noise levels due to the much larger boundary-to-area ratio of the small camps compared to the large tourist camps, and the fact that the tourist camps also have much more of a green buffer zone around the camp. This means that, on average, housing and other sound-generating infrastructure in the ranger camps are much closer to the camp boundaries and less dampened by a green buffer. Moreover, activities in ranger camps are work activities in contrast to the leisure activities in tourist camps, and such work activities may also induce more and louder sounds. Another interesting finding was that the likelihood of rhino presence increased closer to management tracks (in contrast to weak evidence for a negative effect of tourist roads). The fact that management tracks did not deter rhino may be due to these tracks being used less frequently and by much lower traffic volumes than the tourist roads. They also lack the human voice-producing game drive vehicles that frequent the tourist roads. However, instead of a neutral effect on rhino presence, our findings suggest a positive effect of management tracks on rhino presence. One explanation for this could be that areas along management tracks experience increased protection from poaching because of relatively high ranger patrol frequency, leading to higher rhino presence. Increased presence close to management tracks may also reflect the use of these tracks by rhino for longer-distance movement and/or attraction to the tracks’ road verges because of increased grass productivity there, due to water runoff from the road. However, these explanations remain highly speculative and need further testing with more fine-scale data approaches.

We also hypothesized that rhino may learn to avoid long-term poaching hotspots, through surviving rhino frequently encountering dead rhino and/or losing family members in the same area. However, we did not find support for this and found no evidence for an association between poaching density and rhino landscape distribution. The fact that we found no negative relation between poaching hotspots and rhino likelihood of presence suggests that poaching events are too infrequent or spatially unpredictable for rhino to recognize as risky areas. This suggests that poaching hotspots could act as ecological traps: areas that rhino are attracted to without recognizing the risk of increased poaching caused by poachers selecting these areas that attract rhino. Such ecological traps have previously been suggested for Kruger National Park by le Roex et al. (2020). Although not statistically significant, our data indicate a positive association between the likelihood of rhino presence and increased poaching intensity (Fig. 3g). Such a positive association would likely indicate active selection by poachers of areas that rhino select. This is, in fact, what we found for legal removal: a positive association between rhino likelihood of occurrence and intensity of legal removals, likely reflecting the management strategy to remove rhino from high-density areas (Linklater and Shrader 2017). We should also note that the coarse nature of our datasets and analysis approach prevented us from assessing shorter-term (daily, weekly to a few months) and finer-scale (within a few hundred metres) avoidance behaviour of poaching.

## Conclusions: resource availability and human disturbance both drive rhino landscape distribution

Our study is among the first to simultaneously investigate resource and human risk drivers of rhino landscape distribution and space use. Arguably, our most important finding is that both drivers play central roles, evidenced by the fact that our model with both types of drivers explained significantly more variation than our model with only resource drivers. Moreover, as already highlighted earlier, the top three predictors of rhino distribution included both resource and human risk drivers. Terrain ruggedness explained most variation in rhino distribution *(χ*^*2*^ = *250)*, followed by distance to the boundary fence *(χ*^*2*^ = *131)* and catena position (*χ*^*2*^ = *95*)*.* This finding is important and clearly argues for including human risk and disturbance variables in understanding the ecology of this megaherbivore. Our findings thus challenge prevailing thinking that resource drivers are the most important in understanding megaherbivore ecology and behaviour. More specifically, the fact that rhino avoided the reserve boundary, but also smaller and larger camps, strongly suggests the existence of a reserve edge effect (Woodroffe and Ginsberg 1998). Such edge effects are essential to include when planning effective conservation areas for species such as rhino. Such edge effects are likely to be stronger in reserves where human settlements and activities are close to the border (as in iMfolozi) and reduced if the landscape outside the reserve is similar to that inside the reserve (Woodroffe and Ginsberg 1998). Therefore, buffer zones may be necessary to maintain the functional value of reserves that are negatively influenced by edge effects (Woodroffe and Ginsberg 1998). An alternative, or additional, explanation of why rhino avoid the fenceline could be that fences could lead to intensified use of areas along fencelines and localized resource depletion in these areas, as has been shown for elephants (*Loxodonta africana*) (Loarie et al. 2009) and suggested for other large grazing mammals (Gadd 2011), potentially making these areas less attractive to rhinos due to increased resource competition.

## Supplementary Information

Below is the link to the electronic supplementary material.

Supplementary file1 (DOCX 266 KB)

## Data Availability

The rhino aerial count and poaching incident datasets are highly sensitive, and we received official access to the data, and permission to analyse the data for the purpose of this published study, through a formal research application process with the study area’s management organization Ezemvelo KZN Wildlife. This permission was granted under the research project permit number E/5191/05. This permit explicitly states that we are not allowed to publish or publicly share the original datasets. The original data can be accessed by applying to Ezemvelo KZN Wildlife.

## Code availability

https://zenodo.org/records/17036993
