# Supplementary material for: Resource availability and human activity shape the landscape distribution of white rhinoceros, a highly threatened African megaherbivore
Source: Oecologia. 2026 Feb 5;208(2):31. doi: 10.1007/s00442-025-05845-7 (PMC12876089; doi:10.1007/s00442-025-05845-7)
Supplement: Supplementary file 1 — Supplementary file1 (DOCX 266 KB) [file 442_2025_5845_MOESM1_ESM.docx]

**Appendices**

*Table 7. Estimate table from the model with low poaching data.*

|  | **Estimate** | **Std. Error** | **z-value** | **p-value** |
| --- | --- | --- | --- | --- |
| **Intercept** | -2.919 | 0.081 | -35.123 | <0.001*** |
| Distance to Big Camps | 0.000 | 0.000 | 2.087 | 0.036* |
| Distance to Small Camps | 0.000 | 0.000 | 2.676 | 0.007** |
| Distance to Management Tracks | 0.000 | 0.000 | -0.015 | 0.110 |
| Distance to the Boundary Fence | 0.000 | 0.000 | 7.383 | <0.001*** |
| Distance to Road | 0.000 | 0.000 | 3.447 | <0.001*** |
| Poaching Intensity | 0.139 | 0.081 | 1.72 | 0.085 |
| Legal Removal Intensity | 0.328 | 0.098 | 3.353 | <0.001*** |
|  |  |  |  |  |
|  |  |  | AIC: 24120.42 |  |

*Table 8. Estimate table from the model with high poaching data.*

|  | **Estimate** | **Std. Error** | **z-value** | **p-value** |
| --- | --- | --- | --- | --- |
| **Intercept** | -2.927 | 0.083 | -35.087 | <0.001*** |
| Distance to Big Camps | 0.000 | 0.000 | 1.872 | 0.061 |
| Distance to Small Camps | 0.000 | 0.000 | 2.339 | 0.019* |
| Distance to Management Roads | 0.000 | 0.000 | 1.149 | 0.250 |
| Distance to the Boundary Fence | 0.000 | 0.000 | 9.324 | <0.001*** |
| Distance to Road | 0.000 | 0.000 | 0.596 | 0.551 |
| Poaching Intensity | -0.081 | 0.086 | -0.946 | 0.344 |
| Legal Removal Intensity | 0.399 | 0.141 | 2.813 | 0.004** |
|  |  |  |  |  |
|  |  |  | AIC: 20008.36 |  |

*Table 9: Anova table for the model with low poaching data.*

|  | **Chisq.** | **Df** | **p-value** |
| --- | --- | --- | --- |
| Distance to Big Camps | 4.464 | 1 | 0.034* |
| Distance to Small Camps | 4.884 | 1 | 0.027* |
| Distance to Boundary Fence | 53.097 | 1 | <0.001*** |
| Distance to Road | 12.020 | 1 | <0.001*** |
| Poaching Intensity | 2.265 | 1 | 0.132 |
| Legal Removal Intensity | 9.715 | 1 | 0.001** |

*Table 10: Anova table for the model with high poaching data.*

|  | **Chisq.** | **Df** | **p-value** |
| --- | --- | --- | --- |
| Distance to Big Camps | 3.503 | 1 | 0.061 |
| Distance to Small Camps | 5.471 | 1 | 0.019* |
| Distance to Management Roads | 1.319 | 1 | 0.250 |
| Distance to Boundary Fence | 86.932 | 1 | <0.001*** |
| Distance to Road | 0.355 | 1 | 0.551 |
| Poaching Intensity | 0.894 | 1 | 0.344 |
| Legal Removal Intensity | 7.912 | 1 | 0.005** |


*Table 11: Table summarising the VIF values for the numerical predictor variables.*

*
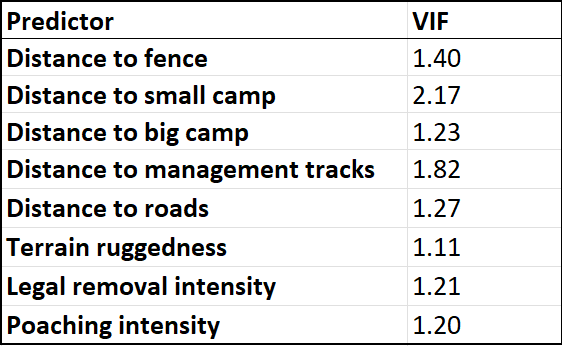
*

*
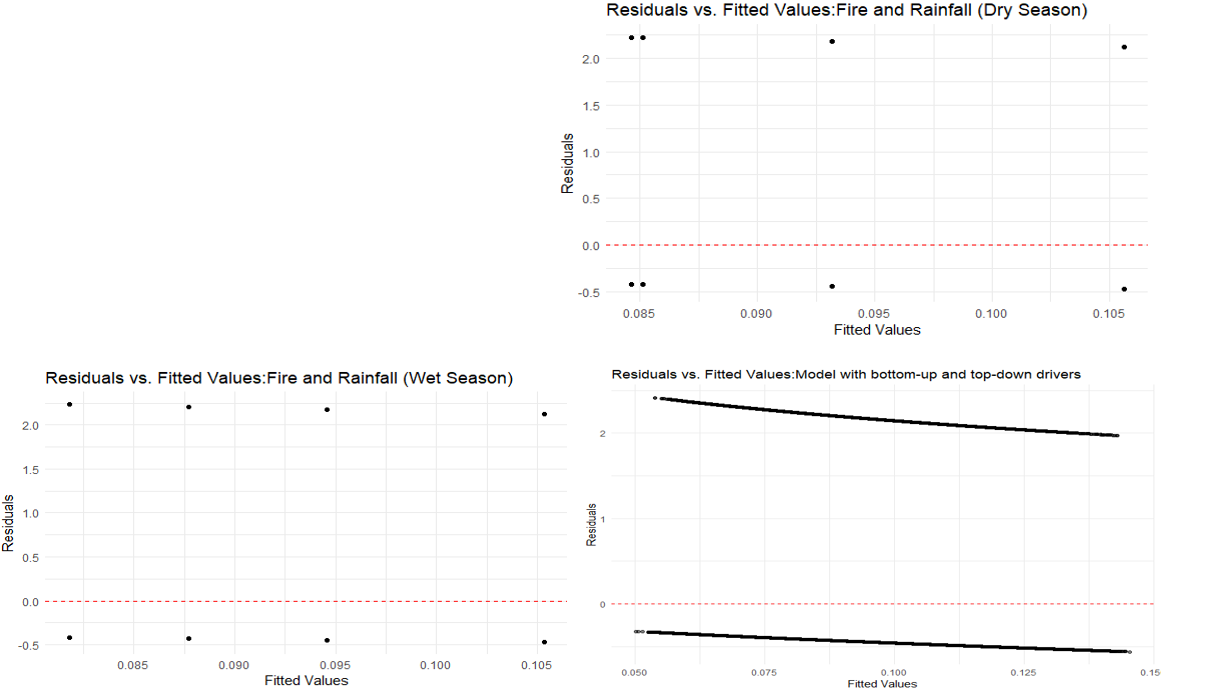
Figure 5: Residuals versus fitted values for the models.*
